# Burnout Among Healthcare Workers in the COVID 19 Era: A Review of the Existing Literature

**DOI:** 10.3389/fpubh.2021.750529

**Published:** 2021-10-29

**Authors:** Carlo Giacomo Leo, Saverio Sabina, Maria Rosaria Tumolo, Antonella Bodini, Giuseppe Ponzini, Eugenio Sabato, Pierpaolo Mincarone

**Affiliations:** ^1^Institute of Clinical Physiology, National Research Council, Lecce, Italy; ^2^Institute for Research on Population and Social Policies, National Research Council, Brindisi, Italy; ^3^Institute for Applied Mathematics and Information Technologies “E. Magenes,” National Research Council, Milan, Italy; ^4^Respiratory Diseases Unit, “A. Perrino” P.O., Brindisi, Italy

**Keywords:** COVID-19, burnout, healthcare workers, mental health, public health

## Abstract

In the current period of global public health crisis due to the COVID-19, healthcare workers are more exposed to physical and mental exhaustion – burnout – for the torment of difficult decisions, the pain of losing patients and colleagues, and the risk of infection, for themselves and their families. The very high number of cases and deaths, and the probable future “waves” raise awareness of these challenging working conditions and the need to address burnout by identifying possible solutions. Measures have been suggested to prevent or reduce burnout at individual level (physical activity, balanced diet, good sleep hygiene, family support, meaningful relationships, reflective practices and small group discussions), organizational level (blame-free environments for sharing experiences and advices, broad involvement in management decisions, multi-disciplinary psychosocial support teams, safe areas to withdraw quickly from stressful situations, adequate time planning, social support), and cultural level (involvement of healthcare workers in the development, implementation, testing, and evaluation of measures against burnout). Although some progress has been made in removing the barrier to psychological support to cope with work-related stress, a cultural change is still needed for the stigma associated with mental illness. The key recommendation is to address the challenges that the emergency poses and to aggregate health, well-being and behavioral science expertise through long term researches with rigorous planning and reporting to drive the necessary cultural change and the improvement of public health systems.

## Introduction

Burnout is a psychological syndrome described as a self-reported state of care- or work- related physical and mental stress (1) that induces emotional exhaustion (EE), depersonalization (DP), and a sense of reduced personal accomplishment (PA) (2). It is an unexpected consequence of an *organizational culture* unable to balance the personal identity of the worker with that of the work organization and the social context, and of the consequent continuous mental effort to cope with the perceived friction (3). Burnout was first applied to healthcare workers (HCWs) by Freudenberger in 1974 (4). Due to substantial disagreement in the health literature on what exactly constitutes burnout and therefore on how to measure it (5), there is a great heterogeneity in the prevalence of this phenomenon: Rodrigues and colleagues, in their meta-analysis on different medical resident specialties (2), reported that the overall prevalence of burnout for all specialties was 35.1%; Rotenstein and colleagues, in their meta-analysis on practicing physicians (5), estimate an overall burnout ranging from 0 to 80.5% with pooled prevalence of 21.3% on overall burnout; they also calculate a pooled prevalence of 34.4% on EE, 25.8% on DP, and of 23.5% on PA.

In the recent period due to the coronavirus disease 2019 (COVID-19) pandemic (6) the world is experiencing an unprecedented global public health crisis with a significant strain on the healthcare system. In fact, the very high number of globally confirmed cases (195,266,156) and deaths (4,180,161) (7) and the probable additional “waves” to come as new variants emerge despite increased vaccination coverage (8) are having a serious impact on health systems: rationing or cessation of routine services, repurposing of clinical areas, redeployment of staff to unfamiliar clinical environments, shortage of personal protective equipment, extensive responsibilities, constant risk of complaints for negligence (9, 10) with medical resources and services placed at their maximum capacity due to unprecedented demands, especially for emergency departments (11). Frontline HCWs involved in the management and diagnosis of COVID-19 are more exposed to overwhelming pressure with consequent psychological stress. As referred in recent publications, medical staff report physical and mental exhaustion – due to the ethical dilemmas and moral injuries for the torment of life-or-death decisions required to be made fast and without the support of optimal care protocols, the pain of losing patients and colleagues, and the risk of infection for themselves and their families (12, 13). All these issues are especially true for residents and young HCWs who, as discussed in Zoorob et al. (14), received ever-changing information on protective measures, and are asked to work in services other than their specialty, particularly in frontline situations (15).

With this narrative review, we aim to discuss the magnitude of burnout among HCWs in the COVID-19 era analyzing emerging concepts to grasp the complexity of the problem. In particular we wanted to identify the health professionals exposed to a greater risk, the effects of burn-out on an individual and organization level and how it has been recommended to address this issue. We also wanted to highlight current research gaps that need to be filled so that health systems can be prepared for future challenges.

## Methods

With this broad perspective in mind, to grasp the complexity of the problem, we performed, as also suggested by Greenhalgh and colleagues (16), a narrative review. The of review of literature has been done without date restrictions; it was conducted on MEDLINE/Pubmed, ISI Web of Knowledge, Scopus, and Google Scholar by a multi-disciplinary team of socio-economists, methodologists, healthcare workers. We limited our search to works published in English or Italian and used the following search terms: “healthcare workers,” “physicians,” “residents,” “nurses,” “burnout,” “chronic pain,” “pain syndrome,” “painful disorders,” “stress,” “workloads,” “suicide,” “Covid19,” “coronavirus disease,” “pandemic”. Study inclusion was assessed through visual inspection of abstracts. Forward citation of relevant papers was also adopted to increase the sensitivity of the search process.

### Who Is Affected

Before COVID-19, Rodrigues and colleagues (2) reported an high variability of the prevalence of burnout across specialties: high prevalence (42.5%), when grouping general surgery, anesthesiology, obstetrics and gynecology, and orthopedics; moderate prevalence (29.4%), for internal medicine, plastic surgery and pediatrics; low prevalence (23.5%), for otolaryngology and neurology. Emergency routine results a key determinant of heterogeneity: prevalence is higher among residents from medical specialty schools who deal directly with life-threatening situations and shift overload. Age emerges as a protective factor: burnout levels in physicians tend to decrease with increasing age, possibly due to the more “idealistic” and empathic approach in younger physicians (17).

In the COVID-19 pandemic, more HCWs are facing life-threatening situations, pathogen exposure, and shift overload and other major changes in work organization (9). Moreover, increased supervision and regulation reduced autonomy of HCWs and their time with patients (18). Prevalence of burnout was higher in intensive care units and sub-intensive care wards, and for residents and nurses. In an Italian survey, higher levels of burnout were found in females, in young (aged <30 years) HCWs, in those who frequently change job duties and family habits, and in residents (19).

In a recent systematic review, Prasad and colleagues (20) found higher stress scores in US health organizations among women, black and Latino individuals, hospital workers and nursing assistants, medical assistants, and social workers. Stress and burnout were associated with fear of exposure or transmission, self-reported anxiety/depression, and work overload. The high exposure to risk for female workers may be linked to their predominance in patient-facing roles, gender discrimination, gender expectations in care, and inattention at “double shift” work with high workloads at home. The high exposure to risk for black and Latino HCWs was linked to a greater fear of exposure to COVID-19 due to racial concordance between workers and patients (black and Latino were overrepresented among patients hospitalized with COVID-19) and entry-level positions to their employment that expose them to a direct contact with patients and with few opportunities for advancement. The risk of infection may be higher in low- or middle-income countries due to a very limited access to personal protective equipment (21).

**Figure 1:**
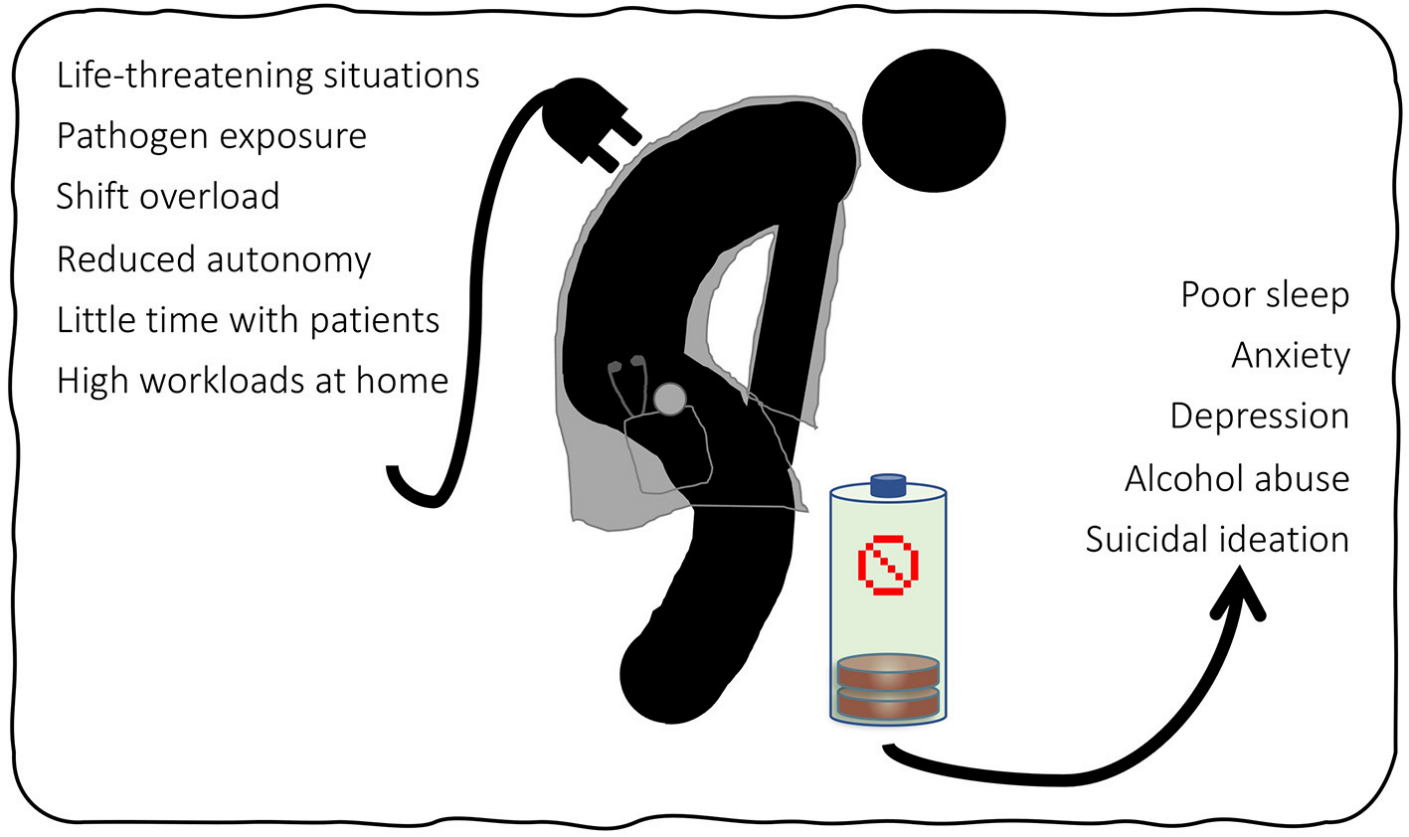
Summary of energy drain factors and consequences of burnout on HCWs.

The above energy drain factors are summarized in Figure 1 along with the consequences of burnout which are discussed in the next section.

### Effects on Healthcare Workers and on Healthcare Systems

A first direct effect of burnout is, of course, on HCWs' own care and safety. The rate of depressive disorder among HCWs is alarming when compared with that of the general population and is closely related to high levels of occupational stress (22). During the COVID-19 outbreak, a relatively high prevalence of anxiety (24.94%), depression (24.83%) and sleep disorders (44.03%) was reported in meta-analyses investigating the mental health of HCWs (23, 24). Healthcare workers tend to hide their difficulties due to the perceived stigma associated with mental illness as well as to the fear of an impact on their careers (25).

In turn, these mental conditions are associated with further criticalities, including a 25% increased odds of alcohol abuse or dependence and a doubled risk of suicidal ideation (18). When considering the extreme act, it is well known that the rates are higher among physicians than in the general populations (26). Dutheil and colleagues (27) recently reported an overall standardized mortality rate for suicide in physicians of 1.44 with an higher level in females of 1.99. They also found a higher risk for anesthesiologists, psychiatrists, general practitioners and general surgeons. Although, at present, no data sets regarding the impact of COVID-19 on physician mental health and suicide are available, the many news published in the newspapers of various countries about the suicide of doctors active in the pandemic leave no doubt that the situation is getting worse (28).

An indirect effect of burnout could be the lowering of the quality of healthcare systems in terms of adherence to guidelines, poor communication, medical errors, and patient outcomes and safety (29). However, as clearly stated by Tawfik and colleagues (30), the relationship between the two phenomena may be bi-directional: HCWs suffering from burnout may not be able to provide high-quality healthcare services, take more unnecessary risks, pay less attention to details, and, conversely, exposure to adverse events or recognition of poor quality of care may lead to psychological distress. The authors conclude that the real strength of the relationship may be less than that reported and that more randomized trials with adequate power and design are needed to understand how exactly burnout and quality of care influence each other.

Burnout is a critical issue that generate inefficiency in healthcare organizations. Shanafelt and colleagues (31) reported that the economic cost of physicians' reduced wellbeing can be mainly assessed in terms of the organizational cost of replacing them, decreased productivity and other “blind” issues. They estimated these costs to be between $ 500,000 and $ 1,000,000 for replacing a single physician with the invaluable training and experience consequently lost. Moreover, they reported a 30% reduction in work effort for each 1-point increase in burnout (on a 7-point scale), and highlighted other costs arising from losing mentors for junior faculty and grants, or from managing medical errors and complaints of negligence.

### How to Address It

Defining strategies to cope with HWC burnout is a relevant research topic regardless of the outbreak of COVID-19 (in the Table 1 we have summarized the measures found that prevent and reduce physician burnout). In 2016, West and colleagues (32) performed a meta-analysis on interventions to prevent and reduce physician burnout. These were focused on both individuals and organizations: facilitating small group curricula, stress management, and training in self-care and communication skills, as interventions on individuals, and shortening the duration of attending rotation and resident shifts, and improvements in clinical work processes, as organizational strategies.

**Table 1:** Measures to prevent and reduce physician burnout.

**Impact level**	**Measures**	**References**
Individual	° Facilitate small group curricula ° plan initiatives for stress management ° train in self-care and communication skills	(32)
	° Stimulate: ∙ physical activity ∙ physical relaxation ∙ balanced diet ∙ good sleep hygiene ∙ family support ∙ small group discussions	(33–36)
Organization	° Shorten the duration of attending rotation and resident shifts ° improve clinical work processes: ∙ limited duration of shifts and on the periods at front line ∙ alternated series of shifts with days off ∙ planned vacations even during an outbreak	(32, 37)
	° Involve all HCWs in management decisions ° structure multi-disciplinary team for professional psychosocial support to HCWs ° compensate HCWs with practical support (social services for child, elderly or animal care)	(37)
	° Promote a principle of co-production with the involvement of patient to share responsibility	(38)
Cultural	° Introduce a radical change in the culture of work by countering the stereotype of endurance, recognizing human limitations on a physical, cognitive and emotional level	(39–42)
	° Adopt a blame-free environment to share incidents, ethical or emergency issues, challenges and advices	(37)

*HCWs, Healthcare workers*.

In addition, Epstein and Privitera (39), called for a radical change in the culture of work by countering the stereotype of endurance which “overvalues stoicism and dismisses complaints as signs of weakness” and recognizing human limitations on a physical, cognitive and emotional level.

Since the COVID-19 outbreak, a great deal of evidence has been generated on burnout in HCWs, leading to extensive discussions on how to address it in this specific context.

Regarding individual measures, self-care is suggested as a line of defense for HCWs to manage requests for assistance of COVID-19 patients, especially when recovery times are short and long-terms efforts are required. Physical activity, physical relaxation, balanced diet, good sleep hygiene, family support, meaningful relationships (also maintained through digital channels), job satisfaction, self-awareness though reflective practices and small group discussions are the reported interventions with evidence of efficacy (33–36).

Based on a scoping review and expert interviews, several recommendations focused on organizations have been proposed to build and maintain the resilience of frontline HCWs exposed to COVID-19 (37). Authors invite to support communication, even during busy periods, by: (a) adopting a blame-free environment to share incidents, ethical or emergency issues, and challenges and advices; (b) involving nurses in management decisions (to promote a sense of togetherness and positivity where every voice has the opportunity to be heard); (c) allowing someone to talk before, during and after a shift. In addition, they recommend structuring a multi-disciplinary team with psychologists, spiritual counselors, social professionals, occupational health and safety physicians for professional psychosocial support to HCWs based on natural coping strategies (acceptance, active coping, positive framing). They also suggest creating a safe area to provide HCWs with the opportunity to quickly withdraw from an emotionally stressful situation and get peer support. Authors propose planning time limitations on the duration of the shifts (distinguishing between day and night, and between light and intense tasks) and on the periods at front line, alternating series of shifts with days off, and planning vacations even during an outbreak. Finally, they report the importance of compensating HCWs with practical support such as social services for child, elderly or animal care.

Regarding the cultural dimension, it was noted that the widely adopted short-term mood boosters that contributed to depict HCWs as “healthcare heroes”, while offering recognitions in the short, can obscure the human needs for support, especially in contexts where mental health is still perceived as a stigma across society (43). Even if some progress has been made in removing the barrier to seeking psychological support in coping with work-related stress (40, 41), a cultural change is still needed for the stigma (42). In our opinion, it is important to promote a principle of co-production (38) which also includes the involvement of patients in the effort to improve healthcare services through their feed-back on the quality and organization of services. Sharing responsibilities allows to reduce the work-related pressures that may lead to burnout.

### Current Research Gap

Research plays a key role in transforming the challenges that the COVID-19 era poses to society, especially healthcare systems, into an opportunity for improvement. Although a large volume of studies since the COVID-19 outbreak have examined the impact of the pandemic on the mental health of HCWs (44), solid evidence on the effectiveness of interventions to support mental well-being during stressful situations is available only from previous healthcare crises and general contexts (45). To adequately address the burnout issue in times of crisis, both large-sized quantitative longitudinal studies and qualitative studies based on first-person reports are needed. These would allow to better understand the impact on mental health of HCWs during and after the pandemic and to identify the best solutions (46). Practice guidelines are needed (47) which also integrate organizational, social, personal, and psychological factors (45). Acceptability, resources, feasibility, long-term sustainability, the impact on patients, and potential harm are reported as additional key themes to be investigated. Due to the difficulty of conducting research during a pandemic, a great heterogeneity and suboptimal designs characterize the current body of evidence. It is advisable, for future researches, to realize rigorous, standardized and transparent protocols for replicability in other settings [better if using reporting standards such as the Template for Intervention Description and Replication, TIDieR (48)], to develop shared definitions of burnout, to use standardized and validated measurement tools and more representative sample sizes, to include follow-up for long-term mental health implications and comparisons with other time periods (5, 44, 45, 49–51). However, one must be aware of the risks of considering only metrics, and health care organizations must focus on process rather than outcome alone, on goals that demonstrate effective improvement of working conditions and not just on achieving a specific threshold score or ranking (52).

## Conclusion

Burnout was a major concern for HCWs since before the COVID-19 pandemic. The current emergency context has added new social and job-related factors that increase the risk of burnout with associated effects on quality of care and efficiency of the system. Based on our knowledge, this is the first work that discusses recently emerging concepts with a comprehensive view. Several measures have been suggested to prevent or reduce this parallel epidemic that calls for action at individual, organizational or cultural level. The key recommendation is to take up the challenges that the emergency imposes and to aggregate competences in health, well-being and behavioral science through rigorously planned and reported long term researches to guide the necessary cultural change and the improvement of public health systems.

## Author Contributions

CL, SS, and PM conceived the work. All authors performed the search and analysis of the literature. CL, SS, and PM wrote the original draft of the manuscript. MT, AB, GP, and ES critically reviewed the manuscript. All authors have read and approved the final version of the manuscript.

## Conflict of Interest

The authors declare that the research was conducted in the absence of any commercial or financial relationships that could be construed as a potential conflict of interest.

## Publisher's Note

All claims expressed in this article are solely those of the authors and do not necessarily represent those of their affiliated organizations, or those of the publisher, the editors and the reviewers. Any product that may be evaluated in this article, or claim that may be made by its manufacturer, is not guaranteed or endorsed by the publisher.
